# Quasi-Static and Dynamic Measurement Capabilities Provided by an Electromagnetic Field-Based Sensory Glove

**DOI:** 10.3390/bios15100640

**Published:** 2025-09-25

**Authors:** Giovanni Saggio, Luca Pietrosanti, I-Jung Lee, Bor-Shing Lin

**Affiliations:** 1 Department of Electronic Engineering, University of Rome Tor Vergata, 00133 Rome, Italy; 2 Department of Computer Science and Information Engineering, National Taipei University, New Taipei City 237303, Taiwan

**Keywords:** data glove, sensory glove, performance assessment, quasi-static performance, dynamic performance

## Abstract

The sensory glove (also known as data or instrumented glove) plays a key role in measuring and tracking hand dexterity. It has been adopted in a variety of different domains, including medical, robotics, virtual reality, and human–computer interaction, to assess hand motor skills and to improve control accuracy. However, no particular technology has been established as the most suitable for all domains, so that different sensory gloves have been developed, adopting different sensors mainly based on optic, electric, magnetic, or mechanical properties. This work investigates the performances of the MANUS Quantum sensory glove that sources an electromagnetic field and measures its changing value at the fingertips during fingers’ flexion. Its performance is determined in terms of measurement repeatability, reproducibility, and reliability during both quasi-static and dynamic hand motor tests.

## 1. Introduction

The human hand is an intricate marvel of anatomy capable of precise movements, as a versatile and essential tool for countless everyday tasks such as grasping, holding, writing, creating, and communicating. Its precision is vital but its complexity makes the measurement of its dexterity a non-trivial task.

Over the past years, different technological approaches have been adopted, based on surface electromyography [1], cameras [2], and bone conduction [3] among others, or based on gloves equipped with sensors, i.e., sensory gloves (SGs) or data, sensor, sensing, sensorized, instrumented gloves. Different SGs have been developed based on different (flexible or rigid) sensors that exploit resistance [4] (ethylene propylene rubber [5], resistive flex sensor [6], strain gauge [6], polymeric hydrogel [7], piezo [8]), capacitance [9], piezoelectricity [10], mechanoluminescence [11], optics (fiber [12], encoder [13]), inertia [14] (combining accelerometers, gyroscopes and magnetometers), magnetism [15], the Hall effect [16], electromyography [17], ultrasound [18], pressure [19], force [20], or some hybrids (e.g., resistance and inertia [21], force and inertia [22], resistance, pressure, and inertia [23]).

In particular, embedded sensors within the glove allow measuring fiber Bragg grating (FBG)-based sensing of the finger joints flexion–extension [24], abduction–adduction [25], and fingertip pressure [26]. As such, SGs can be used to determine the range of motions [27], the hand postures [28], the grip assessments [8], and the pressure exerted [29], and to recognize gestures [30]. These determinations can allow hand function assessments [31], hand pathology evaluation [32]/staging [33]/monitoring [34], teleoperations [35] and telemanipulations [36], musical gestures [37], virtual [38] and computer interactions [39], user-friendly computer-aided design [40], serious game applications [41], data-driven animations [42], sign language recognition [43], arm–hand visual signaling interpretation [44], and beyond.

The wide variety of adoptable sensors and the sheer vastness of applications make it difficult to select the right SG for the particular request. Moreover, the large variety of sensors are mostly adopted for SG prototypes, and only a limited number of SGs are commercially available. Therefore, the selection of the right SG for the particular application requires special attention in considering the necessary accuracy of the SG performances in terms of repeatability, reproducibility, and reliability of the measurements both for quasi-static and dynamic executions of the hand movements.

Overall, here we report the measured performances of an on-the-shelf available SG, electromagnetic field (EMF)-based, termed MANUS Quantum Metaglove (by Manus Technology Group, The Netherlands), in comparison to quasi-static [45] and dynamic [46] performances already reported for two prototypes of SGs, based on resistive flex sensors (RFSs) and on inertial measurement units (IMUs), respectively. As far as we know, this work represents the first to report both quasi-static and dynamic performances obtained for a commercial SG.

## 2. Materials

### 2.1. EMF-Based Glove

**Figure 1:**
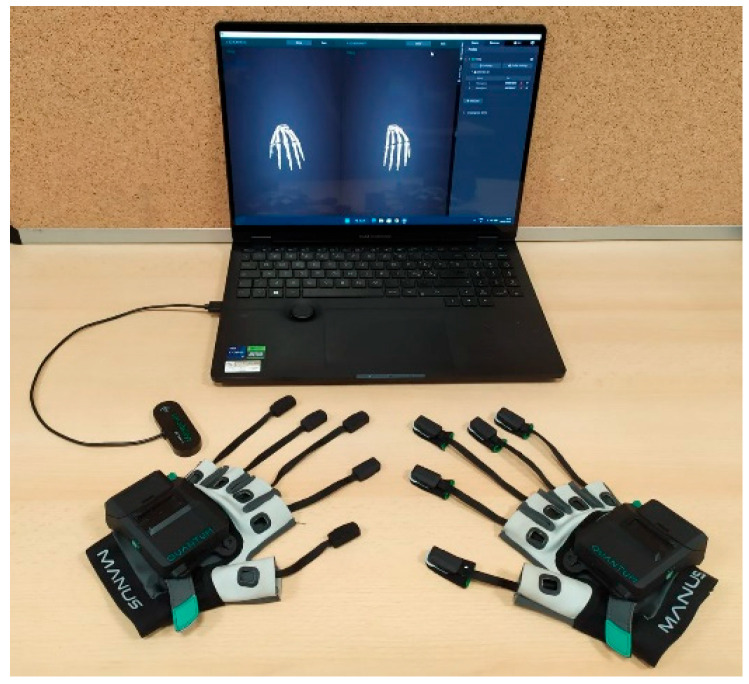
Right and left hands fingerless EMF-based MANUS Quantum Metagloves plus its software for calibrating and data recording.

The MANUS Quantum Metaglove (Figure 1) hosts 1 9-axis IMU and 1 3-axis EMF coil (the source), cased on the dorsal of the hand, and 5 3-axis EMF coils (the detectors) each cased on fingertip sleeves. The source coil generates an EMF and the detectors gather its variations due to fingers’ movements to allow the SG to determine the 6-axis position and rotation of the fingertips by using 9 data combinations between source and detectors. Six degrees of freedom (DoFs) of each fingertip are measured and, by means of a MANUS proprietary calibration and inverse kinematic algorithm, the ranges of motion (RoMs) are approximated for 17 finger joints: carpometacarpal (CMC), metacarpophalangeal (MCP), interphalangeal (IP) joints of the thumb; metacarpophalangeal (MCP), proximal interphalangeal (PIP), distal interphalangeal (DIP) joints of the index, middle, ring, and pinky fingers. Joints with multiple axes of rotation are approximated on multiple axes, joints with a single axis of rotation are approximated on a single axis, obtaining the flexion/extension, adduction/abduction, and reposition/opposition for the thumb, as well as the flexion/(hyper)extension and adduction/abduction for the index, middle, ring, and little fingers. For the hand orientation, the IMU allows measuring the relative rotation, acceleration, and absolute heading for 3-axis rotation of the wrist.

Data communication can be performed in both wired and wireless modes, the former via a USB-C cable, the latter via Bluetooth Low Energy 5 protocol. Furthermore, the MANUS glove is CE marked, guaranteeing compliance with European Union safety, health, and environmental standards, known to be very stringent regarding both safety and electromagnetic compatibility.

### 2.2. Measurement Set-Ups

**Figure 2:**
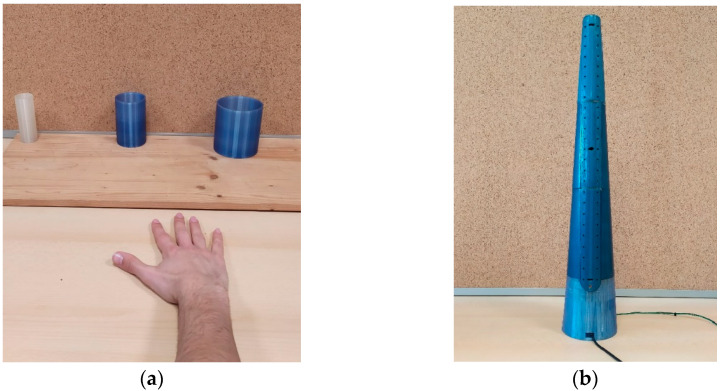
Set-ups for (**a**) quasi-static and (**b**) dynamic assessments of the SG. The three cylinders are 40, 70, and 100 mm in diameter, the cone has a circular base 113 mm in diameter, is 630 mm in height, 35 mm in diameter at the top; the very top LED and the very bottom LED indicate the 40 and 100 mm sections for the limits to follow with hand sliding.

The quasi-static assessments were performed by means of three cylinders of 40, 70, and 100 mm in diameter, respectively (Figure 2a), with the hand gripping them in turn to make the fingers’ joints quasi-statically assume three different repeatable positions [45].

The dynamic assessments were performed by means of a 630 mm height cone with diameters varying from 40 (on top) to 100 mm (at the bottom) (Figure 2b) in order to allow the joints of the fingers to dynamically assume repeatable grasping positions with the hand sliding along its axis [46]. Vertically and equidistantly, 25 pairs of light emission diodes (LEDs) synchronously flashed in sequence, at the start of a push button, together with a synchronized audio beep as feedback for the participant performing the test. Three infra-red (IR) sensors were built into the cone to trace the sliding of the hand. The interval of time between the flashing of two adjacent LEDs was set at 300, 600, and 900 ms, empirically corresponding to fast (~6.94 cm/s), medium (~3.47 cm/s), and slow (~2.31 cm/s) hand movements for three different grasping tests, respectively. The surface of the cone was spray-painted (Isolier und Schutzlack t7, by Teslanol, Wentronic GmbH, Brunswick, Germany) to reduce friction during the hand sliding.

The three cylinders and the cone were realized by 3D printing (printer model X350 Pro, Feldkirchen, Germany).

### 2.3. Participants

According to the literature, six subjects participated in the quasi-static test and six in the dynamic tests (in particular forming two different groups of six to improve in generalization, as reported in [46]). They voluntarily participated, each in turn, restfully seated in front of a table with cylinders and cone placed at hand to minimize motor fatigue during tests, with the upper arm/forearm forming 90°, and the forearm on the table. Moreover, on the tabletop, the hand profile drawing helped the participants to assume a repeatable flat hand position. Table 1 reports the age (years) and the measures of the hand and the palm width (cm) per participant for quasi-static and dynamic tests, respectively.

## 3. Methods

### 3.1. Calibration of the Glove

**Figure 3:**
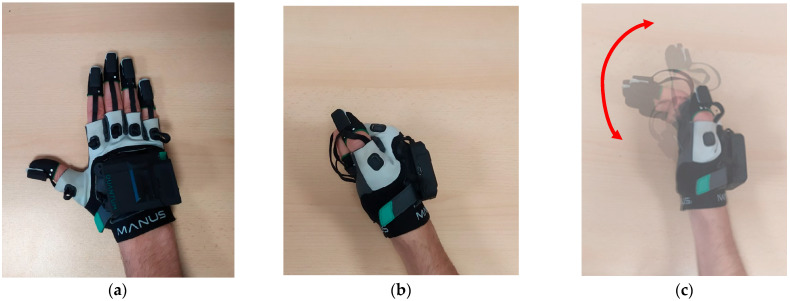
Three steps of calibration: (**a**) flat pose, (**b**) fist pose, (**c**) opening/closing movements.

The calibration of the glove was necessary to record data relative to known positions so to estimate, by means of a MANUS proprietary algorithm through an inverse kinematic approach, the angles of all the fingers’ joints when the hand is assuming whatever posture. The calibration consists in three steps (Figure 3): maintain the hand in a flat position on a tabletop; close the hand with the fingers as close as possible (curled inward); open/close the hand with stretching the fingers along the palm. The second and third steps allowed the software to record the maximum flexion and the radius of curvature assumed by the fingers but with the tips remaining as close as possible or as far as possible, respectively, from the EMF source. The calibration phases do not take into account deformities or pathologies of the hand that could alter the positions and natural trajectories of finger movements, for which it is not valid.

### 3.2. Repeatability and Reproducibility Tests

For each participant, we took into account 14 joints, in particular the angles assumed by two joints of the thumb and three of the other four fingers (MCP, IP, DIP, PIP).

Tests A, Test C, and Test A-dynamic were for repeatability, and Test B, Test D, and Test B-dynamic were for reproducibility of the measurements, respectively, as detailed in the following. Data were acquired at 120 Hz of sampling rate.

#### 3.2.1. Quasi-Static Assessment

**Figure 4:**
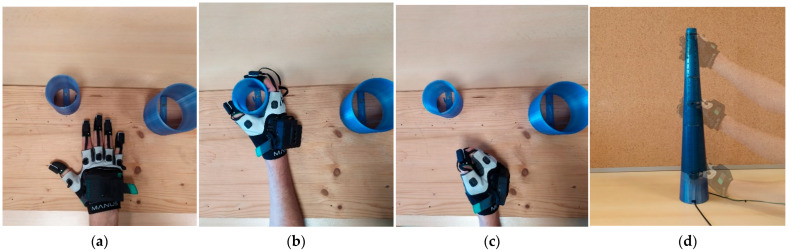
(**a**) Flat pose, (**b**) grip pose for Test A and Test B, (**c**) fist poses for Test C and Test D. (**d**) Hand sliding for dynamic tests.

For the quasi-static assessments, Test A and Test B consisted of posing the hand in a flat position for 6 s and then in a grip position for 6 s, obtaining one trial of data, then repeating the procedure for 10 times, obtaining one block of data, and 10 repetitions of one block formed 10 blocks of data (Figure 4a,b). The flat poses were realized with the hand placed on the surface of a table, with the fingers spread in a comfortable position. The grip poses were realized using each of the three cylinders (so gathering 100 trials each), the participant wrapping the cylinder in turn by hand without using force (in accordance with the method firstly proposed by Wise et al. [47], improved in [27,48,49,50,51], which has now become a standard), and every participant was asked to rest for 3 min between repetitions to avoid fatigue. During Test A, the glove was always on, and for Test B the glove was removed, worn again, and the calibration procedure repeated between each repetition. Accordingly, Test A was for assessing the repeatability, and Test B was for reproducibility of the measures of the angles assumed by the finger joints.

Test C and Test D consisted of 10 repetitions, for 10 blocks of data, of 10 times cycling, for one block of data, of flat and fist poses for 6 secs (Figure 4a,c), for one trial of data, of the hand. The fist poses were realized asking the participants to close the fist by bending the fingers so as to ensure maximum flexion in each joint but without making any effort (again, according to the aforementioned commonly adopted method). The glove was always on during Test C, whilst the glove was doffed, donned, and the calibration refreshed between repetitions during Test D. Accordingly, Test C was for assessing the repeatability, Test D for reproducibility.

#### 3.2.2. Dynamic Assessment

For the dynamic assessments, Test A-dynamic and Test B-dynamic consisted of sliding the hand at the cone surface from top to bottom and vice versa, with minimizing the exerted strength as analogously recommended in [47] for static tests (Figure 4d). To allow controlled and replicable movements, the cone was provided with 25 sync LEDs, which sequentially turn on with programmable timing. The participants were asked to cover with the thumb the sync LED when hand sliding, and the correctness of sliding speed was controlled by three IR proximity sensors placed at the top, center, and bottom of the cone. If the hand was not detected with the proper timing from the IR sensors, measurement stopped and the data discharged.

For Test A-dynamic, one down/up/down scrolling (forming 1 trial of data) was 10 times repeated (forming 1 block of data) and, after 3 min pause to minimize the fatigue, the procedure was repeated three times (forming 3 × 10 blocks of data), with the glove always on, for each time interval (900, 600, 300 ms, respectively) for slow, medium, and fast movements. Test B-dynamic differed from Test A-dynamic only because the glove was removed (off), worn again (on), and recalibrated between two blocks of data. Accordingly, Test A-dynamic was for assessing the repeatability, and Test B-dynamic for reproducibility.

### 3.3. Extraction of the Features

Data acquisition and parameter extraction were performed using home-made routines in MATLAB R2024b (MathWorks Inc., Natick, MA, USA). For the quasi-static test, trials were identified and extracted from each block. Each trial was divided into rest and fist/grip phases and for each phase a sub-interval was obtained by removing phase transition. The mean value of joint angles was computed for each sub-interval phase and then used to compute angle ranges for each block.

Regarding the dynamic measurement, for each trial the time instants at which the subject reached the diameters of 40, 70, and 100 mm were found by means of infrared sensors placed on the cone. Then the main fingers’ joint angles were computed for a time interval around those diameters as in [46].

Trials were identified by *i* (*i* from 1 to 10), blocks by *j* (*j* from 1 to 10), finger joints by *k* (*k* from 1 to 14), and participants by *S* (*S* from 1 to 6).

For quasi-static assessment, data were gathered (and averaged) for the hand in flat, grip, and fist positions, and stored in $X_{ijk}^{flat}$, $X_{ijk}^{grip}$, and $X_{ijk}^{fist}$ matrices, respectively. Further details in [45].

For dynamic assessment, data were gathered (and averaged) at the moments during which the hand was grabbing the cone at the diameters of 40 (top), 70 (middle), and 100 mm (bottom), the thumb covering the LED no. 0, 12, and 24, respectively, both while down/up sliding. Related data were stored in $X_{ijk}^{top}$, $X_{ijk}^{middle}$, and $X_{ijk}^{bottom}$ matrices, respectively. Further details in [46,52].

Assuming ${\bar{X}}_{jk}=\left(1/10\right)\sum_{i=1}^{10}X_{ijk}$, we computed the feature “range” (i.e., measured angles dispersion across trials) for each joint *k* as

(1) $$R_{k}={max}_{j}\left({\bar{X}}_{jk}\right)-{min}_{j}\left({\bar{X}}_{jk}\right),$$

and its associated standard deviation (SD) too. In order to determine the relationships between ranges and SDs, if any, we adopted the Pearson’s product moment correlation coefficient, feature r (the superscript ^¯^ for average) as

(2) $$r=\frac{\sum_{s=1}^{S}\left(R_{s}-{\bar{R}}_{s}\right)\left({SD}_{s}-{\bar{SD}}_{s}\right)}{\sqrt{\sum_{s=1}^{S}{\left(R_{s}-{\bar{R}}_{s}\right)}^{2}}\sqrt{\sum_{s=1}^{S}{\left({SD}_{s}-{\bar{SD}}_{s}\right)}^{2}}}$$

We assessed the reliability too, by calculating the intra-class correlation (ICC), as computed in [48], with adopting the averages of 20 times a random selection of results obtained from two trials out of two random blocks of the *k*-th joint of each participant.

## 4. Results and Discussion

### 4.1. Quasi-Static Tests

**Figure 5:**
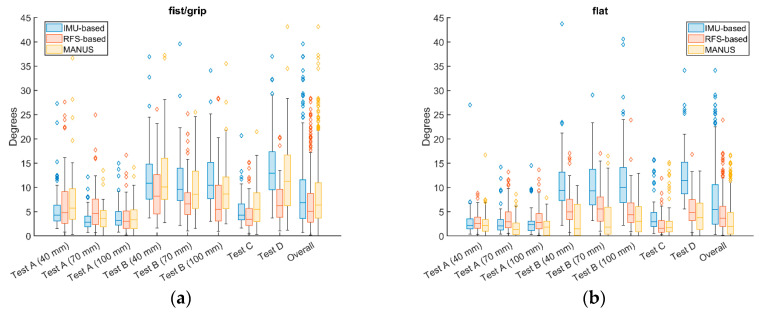
Ranges and average SDs obtained for (**a**) fist/grip and (**b**) flat poses from repeatability and reproducibility tests for the SGs from MANUS, IMU-based, and RFS-based gloves.

Concerning the quasi-static tests, Figure 5 illustrates the averaged (among finger joints) ranges (*Rs*) (in degree) and related standard deviation (SD) values for repeatability (Test A and Test C) and reproducibility (Test B and Test D) of grip/fist and flat poses for the MANUS glove (yellow) in comparison to the RFS-based glove (red) and to the IMU-based glove (blue). Similar behavior results for the three gloves for fist/grip tests (MANUS SG: *Rs* = 3.74–13.32°; IMU-based SG: *Rs* = 3.45–13.89°; RFS-based SG: *Rs* = 3.86–9.33°), whilst the MANUS SG performed slightly better for flat tests (MANUS SG: *Rs* = 1.89–4.34°; IMU-based SG: *Rs* = 2.72–12.76°; RFS-based SG: *Rs* = 2.30–5.88°).

These results are enforced by the Mann–Whitney test results reported in Table 2 (meaningful *p*-value < 0.05 in bold).

**Figure 6:**
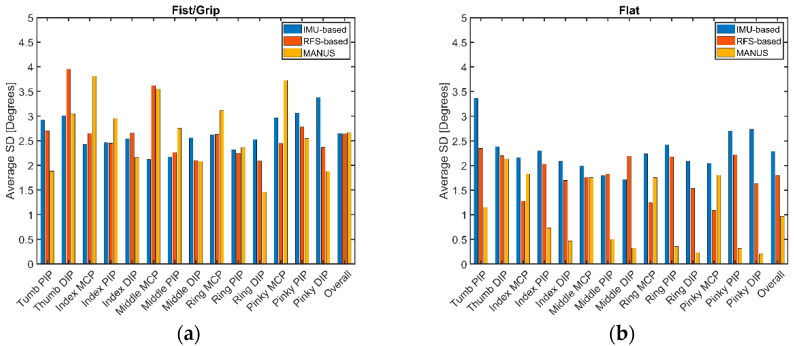
Standard deviation values related to (**a**) fist/grip and (**b**) flat hand poses for the three SGs: MANUS in yellow, IMU-based in blue, and RFS-based in red.

Figure 6 reports the histograms concerning averaged SD values for each finger joint and for each of the three SGs. In particular, for the MANUS glove, SD ranges within 1.4–3.8° for fist/grip and within 0.2–2.1° for flat tests; for the IMU-based glove, SD ranges within 2.12–3.38° for fist/grip and within 1.7–3.36° for flat tests; for the RFS-based glove, SD ranges within 2.09–3.95° for fist/grip and within 1.09–2.35° for flat tests. Some peaks for SDs, e.g., thumb DIP for the RFS-based glove or pinky MPC for the MANUS glove, can possibly be due to differences in grip force exerted by some participants that can to some extent influence the results, as commented in [48]. As expected, the flat poses being more replicable than the fist/grip poses, in general, their related SDs are lower in value. Interestingly, with respect to its SG counterparts (IMU- and RFS-based), the MANUS glove performs in line for fist/grip poses (some slightly worse, e.g., index MCP and other slightly better, e.g., ring DIP), but performs better when measuring flat poses.

**Figure 7:**
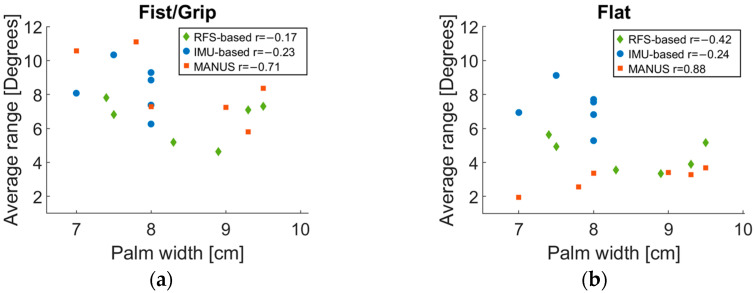
Correlations of range vs. palm width per participants for (**a**) fist/grip and (**b**) flat poses for the MANUS SG.

Figure 7 illustrates the relationships, expressed by means of the feature r, between the palm width and the fist/grip or flat poses for all three SGs. According to the results, no significant correlations were found for RFS- and IMU-based SGs, while for the MANUS SG, a negative correlation (r = −0.71, greater the hand size, lower the measured ranges) was found for fist/grip position, and a positive correlation (r = 0.81, greater the hand size larger the measured ranges) for the flat position. This can probably be due to the aforementioned indirect method used by MANUS to compute joint angles based on a hand model which cannot exactly fit for all hand sizes. However, none of the above-mentioned correlations showed statistical meaning, except for the MANUS glove in the flat position (*p*-value = 0.020). This is probably due to the small sample size. Nevertheless, the mean ranges are in line with those of other SGs for fist/grip results, but lower for flat hand outcomes.

**Figure 8:**
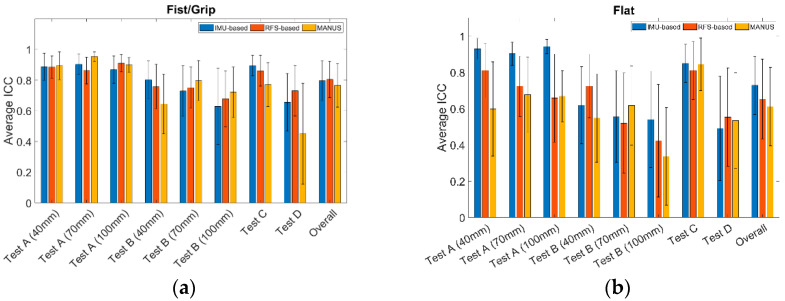
ICC values obtained for (**a**) fist/grip and (**b**) flat position evaluation.

Figure 8 demonstrates the reliability performances of the three SGs in terms of average ICCs and related SDs for all postures. In particular, for grip/fist, the ICCs are within 0.45–0.95, 0.45–0.95, and 0.68–0.91, whilst for flat, the ICCs are within 0.34–0.84, 0.49–0.94, and 0.42–0.81, for the MANUS, IMU-based, and RFS-based glove, respectively. In detail, ICC represents a similitude of measurements for each test, so higher values mean lower dispersion of measured values for a specific test. For the fist/grip measurements, all three SGs perform similarly, with higher ICCs for repeatability tests, and lower for reproducibility with a minimum for Test D (ICC = 0.45). This result is in line with expectations, with reproducibility exhibiting a higher spread of values with respect to repeatability. Regarding the flat position, the RFS-based glove and MANUS SG show lower ICC with respect to the IMU-based counterpart. The overall ICC for the MANUS SG was 0.76 ± 0.14 for fist/grip and 0.61 ± 0.21 for flat pose, for the IMU-based SG, it was 0.79 ± 0.12 for fist/grip and 0.72 ± 0.16 for flat pose, and for the RFS-based SG, it was 0.80 ± 0.11 for fist/grip and 0.65 ± 0.21 for flat pose.

### 4.2. Dynamic Tests

**Figure 9:**
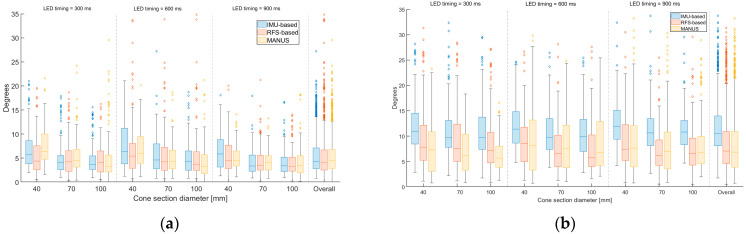
Average ranges for dynamic procedures for different diameters (40, 70, and 100 mm) at different speeds (300, 600, and 900 ms) for (**a**) repeatability and (**b**) reproducibility tests.

For dynamic procedures, Figure 9 shows averaged (among finger joints) ranges (Rs) and related standard deviations (SDs) for slow, medium, and high hand sliding speed, with finger joints measured at the moments when the hand grasped the 40, 70, and 100 mm section (diameter) of the cone, for the MANUS glove (yellow), the RFS-based glove (red), and the IMU-based glove (blue).

As expected, the repeatability shows better results (overall *Rs* within 4.09–7.60 for MANUS SG, 4.06–7.71 for IMU-based SG, 4.16–6.74 for RFS-based glove) with respect to the reproducibility (overall *Rs* within 6.34–9.64 for MANUS SG, 10.61–12.48 for IMU-based SG, 7.39–9.64 for RFS-based glove). Within this frame, the MANUS SG offers performances as good as those obtained for the RFS-based SG, both of which are slightly better than the ones of the IMU-based SG.

These results are enforced by Mann–Whitney test results reported in Table 3 (meaningful *p*-value < 0.05 in bold).

**Figure 10:**
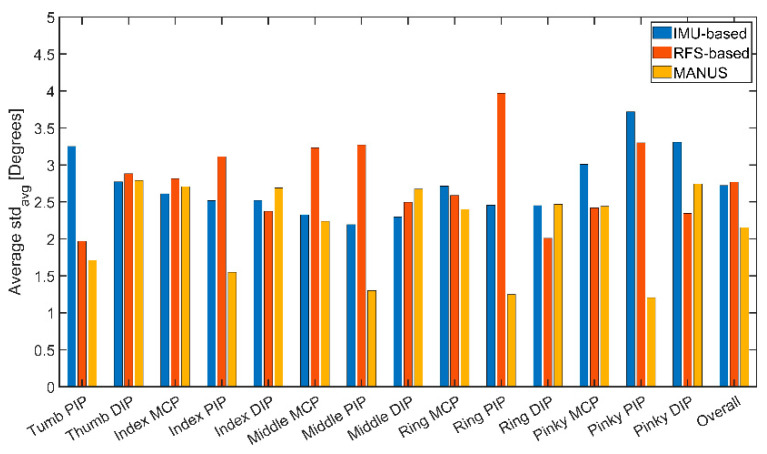
Standard deviation values related to dynamic characterization for the MANUS glove (yellow), the IMU-based glove (in blue), and the RFS-based glove (in red).

Figure 10 shows the values of the average standard deviations (SDs) for the measured ranges (*Rs*) for each measured finger joint for the three SGs. In detail, the MANUS glove demonstrates an SD within 1.20–2.79°, for the IMU-based glove, SD lays within 2.19–3.72°, and for the RFS-based glove, it is between 1.97 and 3.97°. Accordingly, the MANUS glove performs with slightly less dispersion in a dynamic scenario.

**Figure 11:**
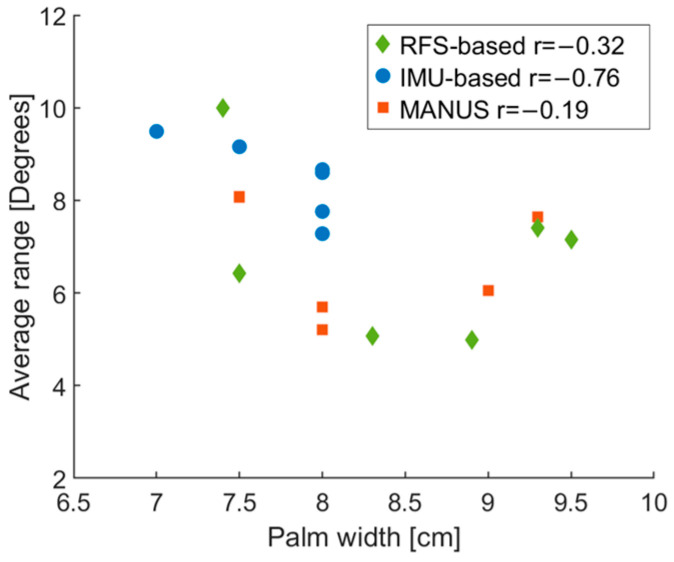
Correlations of range vs. palm width per participants for RFS-based (green), IMU-based (blue), and MANUS glove (orange).

Figure 11 reports correlations between average ranges and palm width for each subject. Specifically, no significant correlation was found for the MANUS (r = −0.19) and the RFS-based (r = −0.32) gloves, while a partial correlation was found for the IMU-based (r = −0.76) glove. However, as in the quasi-static tests, none of the presented correlations reached a significant *p*-value. This result differs from the one obtained for the quasi-static characterization (when a partial correlation was observed).

**Figure 12:**
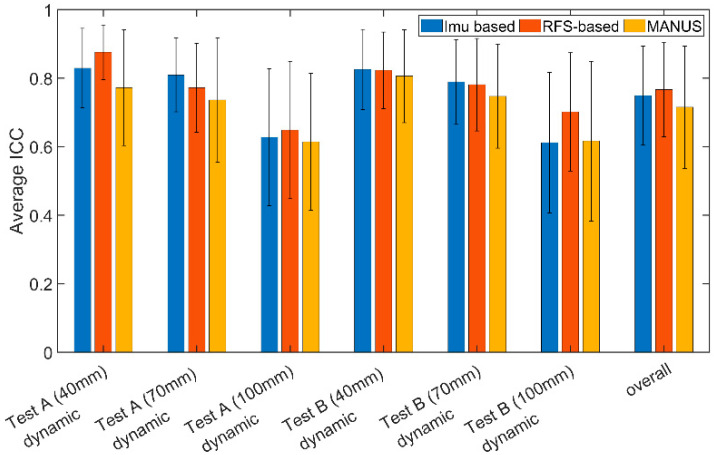
ICC values obtained for dynamic tests for the IMU-based glove (blue), the RFS-based glove (red), the MANUS glove (yellow).

Finally, Figure 12 illustrates the average ICCs and related SDs. In particular, the ICCs are within 0.61–0.81, 0.61–0.83, and 0.65–0.88, for MANUS, IMU-based, and RFS-based gloves, respectively. No particular differences were found between SGs. More into detail, the lowest values are found for the 100 mm diameter cylinder both for repeatability and reproducibility tests, suggesting it is more difficult to obtain similar measurements for the lower angle (flat hand correspond to 0°) for all three SGs. The overall ICC for the MANUS SG was 0.61 ± 0.23, for the IMU-based SG it was 0.61 ± 0.20, and for the RFS-based SG it was 0.70 ± 0.17. These ICC values are slightly lower with respect to the quasi-static tests, suggesting how the three gloves perform better in a static scenario. Overall, the three gloves are characterized by good reliability.

To the best of our knowledge, this is the first study to apply a comprehensive test protocol for evaluating a sensory glove’s repeatability and reproducibility in both static and dynamic conditions. We assessed one commercial glove alongside two prototypes. With interest in sensory gloves on the rise, establishing a standardized evaluation framework is vital; we therefore offer our methodology as a reference for future design and validation efforts. We recognize the protocol’s limitations—most notably, the small participant pool, which restricts the statistical power of correlation analyses. Even so, the high number of trials per test provides sufficient data to render our repeatability and reproducibility results statistically reliable.

## 5. Conclusions

As far as the authors know, this work is the first to report quasi-static and dynamic assessments for an electromagnetic filed-based sensory glove. In particular, we tested the commercially available MANUS Metaglove, by carrying out various quasi-static and dynamic tests involving different subjects with different hand sizes. The assessments were determined by means of repeatability, reproducibility, and reliability scores.

The significance of the results was determined by comparing the performances obtained by the adopted glove with respect to two other sensory gloves, namely an inertial measurement unit-based glove and a resistance flex sensor-based glove, their performances already being reported in the literature.

Regarding the quasi-static tests, the MANUS glove performs in line with the other two gloves when adopting fist/grip poses but better when adopting flat poses. The latter can likely be due to either the null value being less affected by electromagnetic induction error for the MANUS glove, the lower precision around zero flexion for the RFS sensors, or the slightly less stable zero point for the IMU sensors. Moreover, the performances of the MANUS glove can be to some extent correlated to the size of the subject’s hand. Interestingly, it would seem that the negative correlation (for fist) and the positive correlation (for flat) balance each other out in some way with the dynamics.

Regarding the dynamic tests, the MANUS glove performs similar, but with slightly less dispersion, to the RFS-based glove, and both sensory gloves perform slightly better than the IMU-based one. Moreover, the performances of the MANUS glove present no correlation to the subject’s hand size.

The findings indicate that the MANUS glove is ideally suited for applications requiring rapid dynamic response and high precision—especially in medical and rehabilitation contexts (such as those recently explored in [53,54]), where range of motion and movement speed are paramount. Although the sample size of participants aligns with previous studies, further studies can expand that size to increase the statistical power, both to give statistical significance to the differences in hand size correlations in quasi-static and dynamic tests, and to extend it to other SGs based on different technologies.

## Figures and Tables

**Table 1:** Age (years), hand and palm width (cm) of each participant of quasi-static and dynamic tests.

Quasi-Static Test				Dynamic Test			
subject	age	hand	palm	subject	age	hand	palm
S1	29	19.2	9.0	S1	23	17.3	7.5
S2	25	17.0	7.8	S2	29	20.9	9.3
S3	25	20.2	9.5	S3	25	18.5	8.0
S4	29	20.9	9.3	S4	25	17.0	9.0
S5	23	17.0	7.0	S5	25	18.0	7.5
S6	23	20.5	8.0	S6	23	20.5	8.0

**Table 2:** *p*-values of Mann–Whitney comparison test between each pair of SGs for quasi-static tests.

Test	Fist/Grip			Flat		
	Manus vs. IMU	Manus vs. RFS	IMU vs. RFS	Manus vs. IMU	Manus vs. RFS	IMU vs. RFS
Test A (40 mm)	**0.012**	0.126	0.554	0.183	0.062	0.518
Test A (70 mm)	0.453	**0.011**	**0.001**	**<0.001**	**<0.001**	**0.010**
Test A (100 mm)	0.401	0.996	0.348	**0.027**	**<0.001**	**0.014**
Test B (40 mm)	0.850	**0.001**	**0.001**	**<0.001**	**<0.001**	**<0.001**
Test B (70 mm)	0.137	**0.002**	**<0.001**	**<0.001**	**<0.001**	**<0.001**
Test B (100 mm)	**0.006**	**<0.001**	**<0.001**	**<0.001**	**0.003**	**<0.001**
Test C	0.242	**0.004**	**0.027**	**<0.001**	**0.948**	**<0.001**
Test D	0.082	**<0.001**	**<0.001**	**<0.001**	**0.007**	**<0.001**
Overall	0.448	**<0.001**	**<0.001**	**<0.001**	**<0.001**	**<0.001**

**Table 3:** *p*-values of Mann–Whitney comparison test between each pair of SGs for dynamic tests.

		Test A-Dynamic			Test B-Dynamic		
LED timing [ms]	Diameter [mm]	IMU vs. RFS	IMU vs. MANUS	RFS vs. MANUS	IMU vs. RFS	IMU vs. MANUS	RFS vs. MANUS
300	40	**<0.001**	**0.021**	**<0.001**	**<0.001**	**<0.001**	**0.005**
	70	0.886	0.033	0.099	**<0.001**	**<0.001**	**0.001**
	100	0.622	0.115	0.102	**<0.001**	**<0.001**	**<0.001**
600	40	**0.002**	0.223	**0.031**	**<0.001**	**<0.001**	0.524
	70	0.342	0.316	0.897	**<0.001**	**<0.001**	0.327
	100	0.582	**0.002**	**0.009**	**<0.001**	**<0.001**	**0.030**
900	40	0.010	0.024	0.585	**<0.001**	**<0.001**	0.315
	70	0.686	0.281	0.524	**<0.001**	**<0.001**	0.780
	100	0.815	0.999	0.904	**<0.001**	**<0.001**	0.229
	overall	**<0.001**	0.415	**0.015**	**<0.001**	**<0.001**	**0.019**

## Data Availability

Data are available with reasonable requests.

## Abbreviations

The following abbreviations are used in this manuscript:

SGs	Sensory gloves
FBG	Fiber Bragg grating
EMF	Electromagnetic field
RFSs	Resistive flex sensors
IMUs	Inertial measurement units
DoF	Degree of freedom
CMC	Carpometacarpal
MCP	Metacarpophalangeal
IP	Interphalangeal
PIP	Proximal interphalangeal
DIP	Distal interphalangeal
ICC	Intra-class correlation
